# 

**Published:** 1909-10

**Authors:** 


					﻿Sixty .fifth Year. f ,
Vol. LXV.	OCTOBER, 1909. \s.	J
BUFFALO!''^
MEDICALJOURNAL
I 1	IMS by AUSTIN FLINT M. D.
GENERAllUHiLt-!.
> OCT	i ’ EDITOR:
POTTER, M. D.
__	ASSISTANT EDITORS:
WILLIAM C. KRAUSS, M. D.	NELSON W. WILSON, M. D.
ASSOCIATE EDITORS;
JAMES WRIGHT PUTNAM, M. D. ERNEST WENDE, M.D.
JOHN PARMENTER, M. D.	JOHN A. MILLER, Ph. D. ,
MAUD J. FRYE, M. D.
				

